# Application of iridium(III) complex in label-free and non-enzymatic electrochemical detection of hydrogen peroxide based on a novel “on-off-on” switch platform

**DOI:** 10.1038/srep25774

**Published:** 2016-05-12

**Authors:** Xiangmin Miao, Chao Yang, Chung-Hang Leung, Dik-Lung Ma

**Affiliations:** 1 Department of Chemistry, Hong Kong Baptist University, Kowloon Tong, Hong Kong, China; 2 State Key Laboratory of Quality Research in Chinese Medicine, Institute of Chinese Medical Sciences, University of Macau, Macao, China

## Abstract

We herein report a label-free and non-enzymatic electrochemical sensor for the highly sensitive detection of hydrogen peroxide (H_2_O_2_) based on a novel “on-off-on” switch system. In our design, MB was used as an electron mediator to accelerate the electron transfer while AuNPs was used to amplify the electrochemical signal due to its excellent biocompatibility and good conductivity. The “switch-off” state was achieved by introducing the guanine-rich capture probe (CP) and an iridium complex onto the electrode surface to form a hydrophobic layer, which then hinders electron transfer. Upon addition of H_2_O_2_, fenton reaction occurs and produces OH• in the presence of Fe^2+^. The OH• cleaves the CP into DNA fragments, thus resulting in the release of CP and iridium complex from the sensing interface, recovering the electrochemical signal to generate a “switch-on” state. Based on this novel switch system, a detection limit as low as 3.2 pM can be achieved for H_2_O_2_ detection. Moreover, satisfactory results were obtained by using this method for the detection of H_2_O_2_ in sterilized milk. To the best of our knowledge, this is the first G-quadruplex-based electrochemical sensor using an iridium(III) complex.

Hydrogen peroxide (H_2_O_2_) is not only an important compound in food, bioanalysis and environmental analyses^1^, but also plays an important role in cellular reactions catalyzed by many oxidases. Moreover, H_2_O_2_ is also involved in the function and signal transduction of the cell^2^,^3^. Thus, the sensitive and simple detection of H_2_O_2_ is of great interest in chemical, biological, clinical and environmental fields. In the past decades, several kinds of strategies such as electrochemical^4^,^5^,^6^,^7^, fluorescent^8^,^9^,^10^ and colorimetric^11^,^12^,^13^ methods have been developed for the detection of H_2_O_2_. Among these methods, electrochemical sensing has attracted great attention due to its merits such as high sensitivity, rapid response, and simple operation.

Enzyme-based detection methods for H_2_O_2_ have been developed due to number of the advantages of enzymatic reactions, including catalytic activity, high efficiency and good selectivity^14^. Unfortunately, natural enzymes are expensive and easily denatured by environmental changes, thus the enzymes used in these sensing methods are susceptible to potential interfering substances^15^,^16^. Therefore, non-enzymatic electrochemical sensors have received great attention in the development of H_2_O_2_ sensors. Imlay, Linn and co-workers reported that the antibacterial property of H_2_O_2_ is due to DNA damage resulting from Fenton-like reactions in the presence of metal ions such as Fe^2+ ^17, it was reported that H_2_O_2_ could react with certain transition metal ions in low oxidation states and thus produce HO•, which may cleave single-stranded DNA (ssDNA) to DNA fragments^18^. Based on this principle, many biosensors have been developed for biomolecular detection^19^,^20^,^21^.

Guanine-rich (G-rich) nucleic acid sequences, existing in the region of gene promoter and chromosome telomeres, can fold into a unique G-quadruplex structure in the presence of metal ion, such as potassium ion (K^+^)^22^,^23^. Based on these characteristics, many G-quadruplex-based sensors have been developed for the detection of metal ions^24^,^25^,^26^, DNA^27^,^28^, small molecules^29^,^30^ and protein biomarkers^31^. Such K^+^-mediated G-quadruplex-based sensing platforms are convenient, low-cost and relatively quick. Previously, our group has synthesized different iridium(III) complexes and discovered that some of them have highly specific binding properties to G-quadruplex, and a number of detection platforms were subsequently constructed based on the specific interaction between iridium(III) complexes and G-quadruplex DNA^32^,^33^,^34^,^35^,^36^,^37^, through detecting the luminescent enhancement of iridium(III) complexes.

Recently, we discovered that some of the iridium(III) complexes can hinder the electron transfer between the electrode surface and the electrolyte mainly because of the hydrophobicity and the large molecular structure of them. Based on of this unique property, we designed the application of one type of the iridium(III) that specific to G-quadruplex (the structure of it were shown in Fig. 1) in label-free and non-enzymatic electrochemical sensing platform for H_2_O_2_ by using a novel “on-off-on” switch system. As shown in Fig. 1, the first “switch on” state was achieved based on the immobilize of Nafion (Nf) and methylene blue (Nf@MB) composite membrane as inner-layer, and self-assembled positively charged gold nanoparticles ((+)AuNPs) as outer-layer. Successively, the capture probe (CP, guanine-rich nucleic acid) was modified onto the (+)AuNPs surface through an Au-S bond, and the CP would change to G-quadruplex structure in the presence of K^+^, followed by the specific binding with an iridium(III) complex using a simple and label-free method^38^,^39^,^40^,^41^,^42^,^43^,^44^. As a consequence, a hydrophobic and large molecule structural layer wad formed on the electrode surface, and resulted in a “switch off” state. Then, upon incubation of the sensor with H_2_O_2_ and Fe^2+^, Fenton reaction would happen and produced tremendous OH• to cleave CP into DNA fragments, leading to the release of such fragments and the iridium(III) complex from the sensing interface, recovering the electrochemical signal and achieving a “switch on” state. Importantly, the electrochemical signal change was proportional to H_2_O_2_ concentration. Thus, H_2_O_2_ could be detected by using such proposed method based on monitoring the signal change of MB. There are several advantages for such a proposed method: Firstly, due to the good stability of negatively charged Nf membrane on the electrode surface, abundant positively charged and highly stable Nf@MB membrane could formed on the electrode, which can greatly improve the stability of the sensor. In addition, MB is highly conductive as a redox indicator, which can great enhance the sensitivity of the sensor. Meantime, about 4 nm of (+)AuNPs, with excellent biocompatibility and good conductivity, was successfully self-assembly onto the Nf@MB membrane, which can greatly enlarge the electrode surface for more CP immobilization. Moreover, to the best of our knowledge, this is the first application of the iridium(III) complex and (+)AuNPs in electrochemical sensor for H_2_O_2_ analysis. We envision that this “on-off-on” platform could also provide new opportunities for biosensor development.

## Results and Discussion

**Figure 2:**
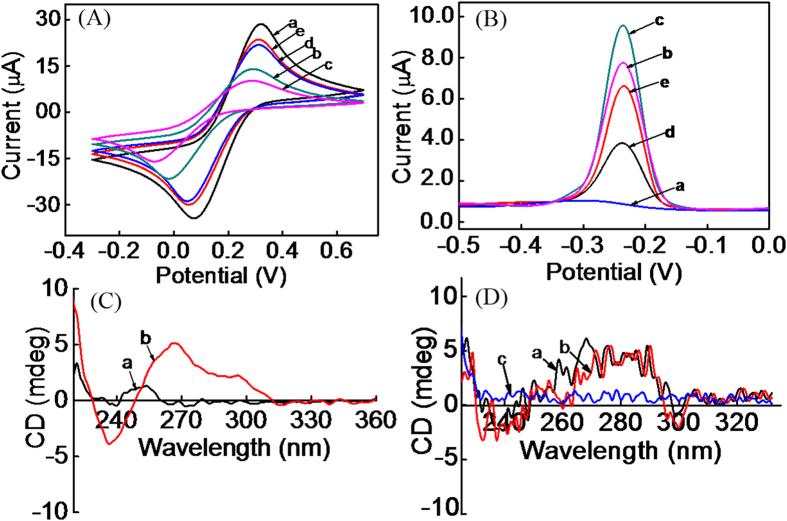
(**A**) Conductivity investigation of the iridium(III) complex and other organic dyes that specific to G-quadruplex in 5 mM of Fe(CN)^3−/4−^: (a) Gold electrode; (b) CP/gold electrode; (c) iridium(III) complex/CP/gold electrode; (d) CV/CP/gold electrode. (e) Rhodamine/CP/gold electrode. (**B**) DPV of the different modified electrodes in 10 mM tris-buffer solution (pH 7.4): (a) Gold electrode; (b) Nf@MB/gold electrode; (c) (+)AuNPs/Nf@MB/gold electrode; (d) iridium(III) complex/CP/(+)AuNPs/Nf@MB/gold electrode; (e) (d) after the incubation with 1.5 nM of H_2_O_2_. (**C**) Circular dichroism of CP before (a) and after (b) the incubation of it with tris-buffer that contained 100 mM of K^+^. (**D**) Circular dichroism of G-quadruplex structure before (a) and after (b) the selective interaction of it with iridium(III) complex, and after the cleavage with H_2_O_2_ (c).

To investigate the conductivity of the iridium(III) complex, several modified electrodes were characterized by cyclic voltammetry experiments (CVs) in the presence of 5 mM Fe(CN)_6_^3−/4−^. As seen from curve a in Fig. 2A, a stable and well-defined redox peak was obtained when the bare gold electrode was scanned in 5 mM of Fe(CN)_6_^3−/4−^. After the immobilization of CP on the electrode surface, an obvious decrease of the peak current was observed, which was attributed to the fact that DNA hinders electron transfer (curve b)^45^,^46^. Then, G-quadruplex structures were formed after the CP-modified electrode was incubated in tris-buffer containing 100 mM of K^+^. Subsequently, the interaction of iridium(III) complex with the G-quadruplex led to a further decrease in peak current, because of the high resistance of the electrode interface induced by the adsorption of iridium(III) complex and the formation of a hydrophobic layer on the electrode surface (curve c). Since other organic dyes such as crystal violet (CV) and rhodamine can also specifically bind to G-quadruplex^47^,^48^, we also investigated the conductivity of CV and rhodamine after they bound to the G-quadruplex that immobilized on the electrode. The results demonstrated that CV and rhodamine would accelerate the electron transfer (curve d and e). Therefore, to obtain the step of the “switch off” state, we selected iridium(III) complex as the hydrophobic layer.

In order to confirm the successful fabrication of the proposed sensing platform, the fabrication process of the sensor was characterized by DPV experiments. As shown in Fig. 2B, no oxidation peak was observed at the bare gold electrode while an obvious oxidation peak appeared after electro-polymerization of MB on the electrode surface, which is mainly due to the high conductivity of MB as an electron mediator (curve b). Then, the peak current increased after the electro-deposition of AuNPs onto the MB film on account of the excellent conductivity of AuNPs (curve c). However, after the interaction of iridium(III) complex with G-quadruplex form of CP in the presence of K^+^, the peak current obviously decreased, since iridium(III) complex would form a hydrophobic and large molecule structural layer on the electrode surface and hinder the electron transfer (curve d). However, upon incubation of the sensor with 1.5 nM of H_2_O_2_ and Fe^2+^, a dramatic increase of the peak current was observed (curve e). This increase could be ascribed to the release of iridium(III) complex and CP fragments from the electrode surface after the cleavage of CP by Fenton reaction in the presence of Fe^2+^ and H_2_O_2_.

To validate the mechanism of the assay, circular dichroism (CD) spectroscopy was performed. As shown in Fig. 2C, there was no obvious peak for CP alone, while a positive Cotton effect peak at around 264 nm and a negative Cotton effect peak at around 236 nm appeared after CP was incubated with tris-buffer containing 100 mM of K^+^, indicating the formation of the classic G-quadruplex structure in accordance with literature^49^. No significant change was observed upon the selective interaction of the iridium(III) complex with the G-quadruplex structure of CP (Fig. 2D, curve a and b). However, the intensity at the peak of 264 nm and 236 nm decreased dramatically when the CP was incubated with H_2_O_2_, these results might be attributed to the cleavage of CP (curve c).

**Figure 3:**
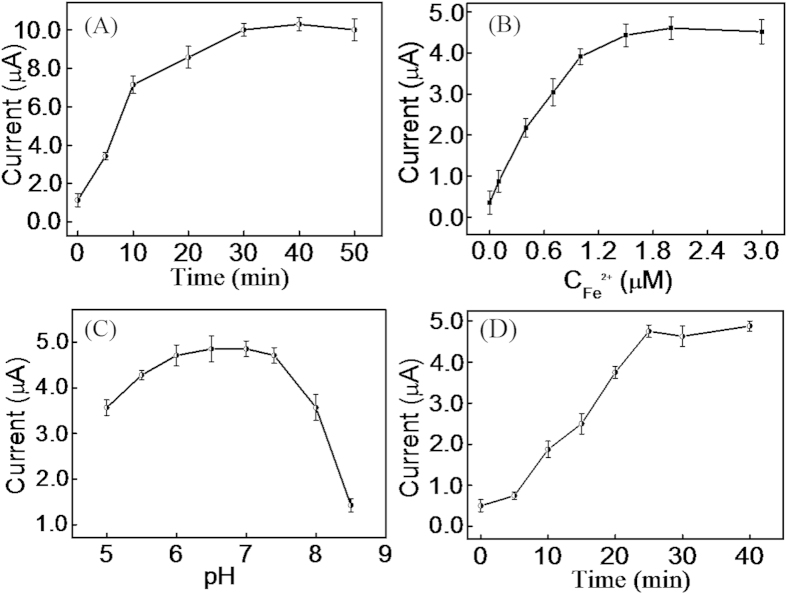
Effect of the self-assembly time of (+)AuNPs on the sensor properties (**A**) Effect of the concentration of Fe^2+^ (**B**), the pH value (**C**) and the incubation time for CP cleavage in the presence of 1.2 nM H_2_O_2_ (**D**).

Experimental conditions including the self-assembly time of (+)AuNPs, the concentration of Fe^2+^ and the cleavage time of CP by H_2_O_2_ were optimized. The self-assembly time of (+)AuNPs is an important factor that might affect the properties of the sensor. As shown in Fig. 3A, the current intensity was proportional to the self-assembly time of (+)AuNPs from 0 to 30 min and then reached a plateau. In view of the sensitivity of the sensor, an electro-deposition time of 30 min was chosen for all the experiments.

The role of Fe^2+^ in the Fenton reaction is to catalyze the cleavage of DNA. Thus, Fe^2+^ concentration is an important factor that can affect the degree of CP cleavage. Figure 3B shows that the current signal increased along with the increase of Fe^2+^ concentration up to 1.5 μM in the presence of 1.2 nM H_2_O_2_, indicating that CP could be cleaved effectively upon addition of 1.5 μM of Fe^2+^. Thus, we selected 1.5 μM as the optimal concentration of Fe^2+^ in the experiments.

DNA structure depends on the pH environment, so the cleavage activity of H_2_O_2_ and Fe^2+^ may depend on the pH as well. As shown in Fig. 3C, the current signal increased with the pH over the range from 5.0 to 6.5 in the absence of H_2_O_2_, and reached a plateau over the range of 6.5–7.4. However, when the pH was higher than 7.4, the current signal decreased dramatically, such result might be due to the fact that an acidic environment can stabilize the Fe^2+^ions, and higher pH can induce the oxidation of ferrous ions to ferric ions under atmosphere (O_2_). Considering the sensitivity of the sensor, we selected 7.4 for further experiments.

Since the second “switch-on” state depends on the cleavage of CP, the cleavage time of CP in the presence of 1.2 nM H_2_O_2_ was investigated in Fig. 3D. It was found that the current signal increased when the cleavage time increased from 0 to 25 min, and then reached a plateau. To achieve the effective cleavage of CP, 30 min was chosen as the cleavage time of CP.

**Figure 4:**
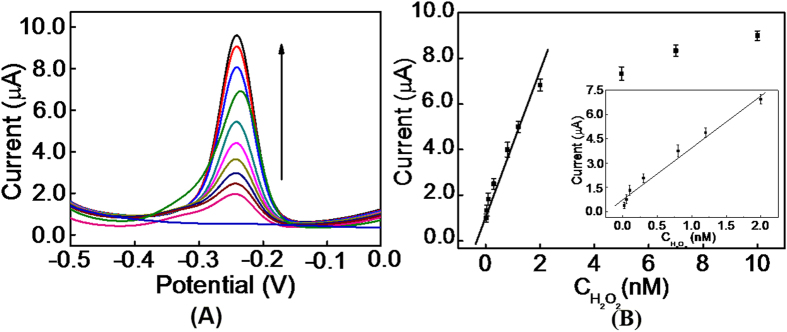
(**A**) DPV experiments for H_2_O_2_ detection in 10 mM of tris-buffer solution (pH 7.4); (**B**) Calibration curve for the sensor.

To evaluate the effect of H_2_O_2_ concentration on current signal, the proposed sensor was incubated with tris-buffer that contained different concentration of H_2_O_2_. As shown in Fig. 4A, the DPV signal increased along with the increase of H_2_O_2_ concentration. According to the calibration plot of Fig. 4B, the linear regression equation was I = 11.18 + 7.87c (c: nM) in a dynamic range from 8.0 pM to 2.0 nM with a correlation coefficient of R^2^ = 0.994. The detection limit was 3.2 pM calculated by the three-signal method. Here, the electro-deposition of AuNPs onto MB film can greatly enlarge the electrode surface for CP immobilization, and accordingly improve the sensitivity of the sensor. The detection limit of our “on-off-on” detection platform for H_2_O_2_ detection was lower than other AuNPs^49^,^50^,^51^,^52^,^53^ or MB^54^,^55^,^56^,^57^ based electrochemical methods, which can satisfy the demand for H_2_O_2_ detection in real samples such as food or industrial products. Thus, our proposed sensor could be used to quantify H_2_O_2_ for food safety and clinical diagnosis.

**Figure 5:**
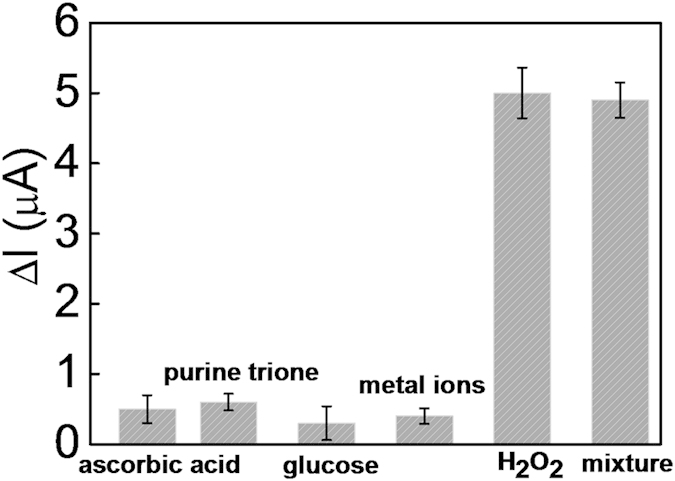
Selectivity of the sensor for H_2_O_2_ (1.5 nM) detection against interferences including ascorbic acid (150 nM), purine trione (150 nM), glucose (150 nM) and metal ions (150 nM).

The stability of the sensor was investigated by CV. After continuous scanning for 20 cycles, the sensor retained 98.1% of its initial response, indicating acceptable stability. Meantime, the selectivity of such sensor for H_2_O_2_ was evaluated against other interferences including ascorbic acid, purine trione, glucose and mixed metal ions (contained Pb^2+^, Cu^2+^, K^+^, Ca^2+^ and Mg^2+^). From the results in Fig. 5, it could be seen that the current intensity change (ΔI) upon addition of ascorbic acid (150 nM), purine trione (150 nM), glucose (150 nM) and metal ions (150 nM of Pb^2+^, Cu^2+^, K^+^, Ca^2+^ and Mg^2+^) were much lower than that of H_2_O_2_ (1.5 nM). Moreover, the ΔI of the sensor after the incubation of it with a solution containing both H_2_O_2_ and the interferences was almost the same with that of H_2_O_2_ (1.5 nM) only. Such high selectivity could be attributed to the highly specific cleavage of DNA based on the Fenton reaction in the presence of H_2_O_2_ and Fe^2+^.

H_2_O_2_ is used as a stabilizer in milk in some European and American countries. However, residual H_2_O_2_ may cause adverse effects to the human body. Thus, the sensitive and rapid detection of H_2_O_2_ in milk samples is of great importance. To evaluate the robustness of the system for H_2_O_2_ detection in milk, milk samples were collected and centrifuged at 13600 rpm to remove fat. After corroborating the absence of hydrogen peroxide, such milk samples were diluted ten-fold with tris-buffer (pH 7.4) for recovery experiments. As shown in Table 1, a good recovery ranging from 96.0% to 109% and a relative standard deviation (RSD) between 1.38% and 3.16% were obtained (each sample was tested 3 times). This result indicated that our sensor has potential application for the quantitative determination of H_2_O_2_ level in real samples.

In conclusion, a novel and label-free “on-off-on” switch system was successfully developed for the sensitive detection of H_2_O_2_. The first “switch on” state was achieved based on the immobilization of Nf@MB and (+)AuNPs. Here, MB was used as the current signal and (+)AuNPs were used to enlarge the surface of the electrode and improve the sensitivity of the sensor. Then, the “switch off” state was obtained based on the formation of a massive hydrophobic layer that contained G-quadruplex structures and iridium(III) complex on the electrode surface. After the incubation of the sensor with H_2_O_2_, CP would be cleaved into DNA fragments and released from the electrode surface, followed by signal recovering as the second “switch on” state. The novel concept of this “on-off-on” platform was successfully used for H_2_O_2_ detection with a detection limit down to 3.2 pM. Moreover, experiments proved that such strategy could be applied effectively for H_2_O_2_ detection in milk samples.

## Methods

### Materials and reagents

Nafion, Methylene blue (MB), rhodamine, thiazole orange (TO), 6-mercapto-1-hexanol (MCH) and gold chloride (HAuCl_4_) were purchased from Sigma Aldrich (St. Louis, MO). The milk sample (Hokkaido Specially Select 3.6 Milk, UHT Processed whole milk, fat: 4.2 g per 100mL, carbohydrates: 4.8 g per 100 mL, sodium: 41 mg per 100 mL, calcium: 115 mg per 100 mL) was purchased from supermarket in Hong Kong. The iridium(III) complex used in the experiment that specific to G-quadruplex DNA was selected and synthesized according to our previous method^58^. Other chemicals and solvents were of analytical grade and double distilled water was used throughout this study. 10.0 mM of tris-buffer (pH 7.4) was used for H_2_O_2_ detection and 10.0 mM of tris-buffer (pH 7.4, with 100 mM K^+^) was used for G-quadruplex formation. The capture probe (CP) was designed according to the literature based on the principle that there are preferential sequences for iron-mediated DNA cleavage. CP was synthesized by Techdragon Inc. (Hong Kong, China) with the sequence of 5′-GTG_3_TAG_3_CG_3_T_2_G_2_-(CH_2_)_6_-SH-3′.

### Apparatus

Electrochemical measurements were monitored by a CHI 630C electrochemical workstation (CH Instruments, Inc. U.S.A.). A conventional three-electrode system consisted of a modified working electrode, a platinum wire counter electrode and an Ag/AgCl reference electrode was used in the experiment. CD spectroscopy measurements were constructed by using an Olis 17/UV/VIS/NIR spectropolarimeter at room temperature.

### H_2_O_2_ sensing protocol

Gold electrodes were pretreated according to our previous method. Then, 0.5 mg of MB was added into 2 mL of 0.5% Nf ethanol solution and sonicated to obtain a homogeneous suspension contained Nf@MB. Next 5 μL of Nf@MB was cast on the pretreated electrode and dried in the air to obtain an Nf@MB film. After that, 4 nm of (+)AuNPs prepared according to literature^59^ was self-assembly onto the Nf@MB film for 30 min, based on the electrostatic adsorption to enlarge the electrode surface for CP immobilization, Subsequently, the modified electrodes were incubated in 10 mM of tris-buffer (pH 7.4) that contained 2.0 μM of CP for 12 h at room temperature (prior to modification, the disulfide bond at the 3′ end of CP was cleaved with tris(2-carboxyethyl)phosphine (TCEP)), followed by reaction with 2.0 μM of MCH for 1 h to block nonspecific sites, and incubated in 10 mM of tris-buffer (pH 7.4) that contained 100 mM of K^+^ and 3.0 μM of iridium(III) complex for another 1 h to induce the formation of a hydrophobic layer on the electrode surface. Then, the prepared sensor was incubated with different concentrations of H_2_O_2_ at 37 °C for 30 min in the presence of Fe^2+^. Finally, the electrochemical characteristics of the sensor were investigated in tris-buffer by using differential pulse experiments (DPV) from −500 mV to 0.0 mV at room temperature.

## Additional Information

**How to cite this article**: Miao, X. *et al.* Application of iridium(III) complex in label-free and non-enzymatic electrochemical detection of hydrogen peroxide based on a novel "on-off-on" switch platform. *Sci. Rep.* **6**, 25774; doi: 10.1038/srep25774 (2016).

## Figures and Tables

**Figure 1:**
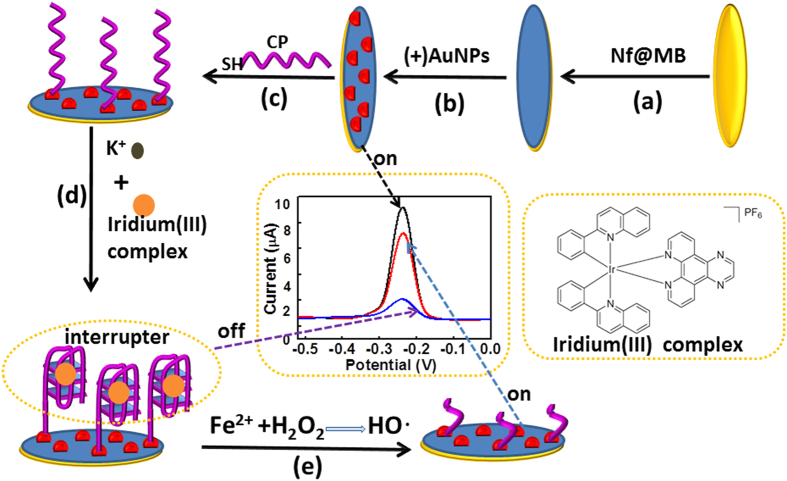
Scheme diagram for the preparation of H_2_O_2_ sensor.

**Table 1:** Recovery experiments for H_2_O_2_ in milk samples.

Sample	Added (nM)	Found (nM)	Recovery %	RSD %
	0.10	0.11	110	2.06
Milk	0.30	0.29	96.7	1.38
	0.50	0.48	96.0	3.16
	1.00	1.09	109	2.12

## References

[b1] Baghayeri M., Zare E. N. & Lakouraj M. M. A simple hydrogen peroxide biosensor based on a novel electro-magnetic poly(*p*-phenylenediamine)@Fe_3_O_4_ nanocomposite. Biosens. Bioelectron. 55, 259–265 (2014). 24389389 10.1016/j.bios.2013.12.033

[b2] Chen X. *et al.* Advances in enzyme-free electrochemical sensors for hydrogen peroxide, glucose, and uric acid. Microchim. Acta 181, 689–705 (2014).

[b3] Terai T. & Nagano T. Small-molecule fluorophores and fluorescent probes for bioimaging. Arch. Eur. Phys. 465, 347–359 (2013). 10.1007/s00424-013-1234-z 23412659

[b4] Liu Q. *et al.* NiO nanoparticles modified with 5,10,15,20-tetrakis(4-carboxyl pheyl)-porphyrin: Promising peroxidase mimetics for H_2_O_2_ and glucose detection. Biosens. Bioelectron. 64, 147–153 (2014). 25212068 10.1016/j.bios.2014.08.062

[b5] Zhang Y. *et al.* Fabrication of 2D ordered mesoporous carbon nitride and its use as electrochemical sensing platform for H_2_O_2_, nitrobenzene, and NADH detection. Biosens. Bioelectron. 53, 250–256 (2014). 24144555 10.1016/j.bios.2013.10.001

[b6] Lin S. *et al.* Luminescence switch-on detection of protein tyrosine kinase-7 using a G-quadruplex-selective probe. Chem. Sci. 6, 4284–4290 (2015). 10.1039/c5sc01320h PMC5707507 29218197

[b7] Wu S. H. *et al.* Amplified electrochemical hydrogen peroxide reduction based on hemin/G-quadruplex DNAzyme as electrocatalyst at gold particles modified heated copper disk electrode. Biosens. Bioelectron. 73, 41–46 (2015). 26043314 10.1016/j.bios.2015.05.039

[b8] Bortolozzi R., Gradowsk S. & Ihmels H. Selective ratiometric detection of H_2_O_2_ in water and in living cells with boronobenzo[***b***]quinolizinium derivatives. Chem. Commun. 50, 8242–8245 (2014). 10.1039/c4cc02283a 24938373

[b9] Marks P. *et al.* Highly efficient detection of hydrogen peroxide in solution and in the vapor phase ***via*** fluorescence quenching. Chem. Commun. 51, 7061–7064 (2015). 10.1039/c5cc01105a 25806424

[b10] Chang H. C. & Ho J. A. Gold nanocluster-assisted fluorescent detection for hydrogen peroxide and cholesterol based on the inner filter effect of gold nanoparticles. Anal. Chem. 87, 10362–10367 (2015). 26379119 10.1021/acs.analchem.5b02452

[b11] Ge S. G. *et al.* Colorimetric detection of the flux of hydrogen peroxide released from living cells based on the high peroxidase-like catalytic performance of porous PtPd nanorods. Biosens. Bioelectron. 71, 456–462 (2015). 25982545 10.1016/j.bios.2015.04.055

[b12] Chen S. *et al.* *In situ* growth of silver nanoparticles on graphene quantum dots for ultrasensitive colorimetric detection of H_2_O_2_ and glucose. Anal. Chem. 86, 6689–6694 (2014). 24862345 10.1021/ac501497d

[b13] Wang G. L. *et al.* Ultrasensitive and dual functional colorimetric sensors for mercury(II) ions and hydrogen peroxide based on catalytic reduction property of silver nanoparticles. Biosens. Bioelectron. 31, 337–342 (2012). 22093771 10.1016/j.bios.2011.10.041

[b14] Privett B. J., Shin J. H. & Schoenfisch M. H. Electrochemical sensors. Anal. Chem. 82, 4723–4241 (2010). 20476724 10.1021/ac101075n PMC3566637

[b15] Zhang B. *et al.* Anodic-stripping voltammetric immunoassay for ultrasensitive detection of low-abundance proteins using quantum dot aggregated hollow microspheres. Chem. Eur. J. 19, 2496–2503 (2013). 23292875 10.1002/chem.201203131

[b16] Gao Z. *et al.* High-resolution colorimetric assay for rapid visual readout of phosphatase activity based on gold/silver core/shell nanorod. ACS Appl. Mater. Interfaces, 6, 18243–18250 (2014). 25244147 10.1021/am505342r

[b17] Imlay J. & Linn S. DNA damage and oxygen radical toxicity. Science 240, 1302–1309 (1988). 3287616 10.1126/science.3287616

[b18] Imiay J., Chin S. & Linn S. Toxic DNA damage by hydrogen peroxide through the Fenton reaction *in vivo* and *in vitro*. Science 240, 640–642 (1988). 2834821 10.1126/science.2834821

[b19] Li H. & Rothberg L. Colorimetric detection of DNA sequences based on electrostatic interactions with unmodified gold nanoparticles. Proc. Natl. Acad. Sci. USA 101, 14036–14039 (2004). 15381774 10.1073/pnas.0406115101 PMC521116

[b20] Huang W. T. *et al.* A simple and facile strategy based on Fenton-induced DNA cleavage for fluorescent turn-on detection of hydroxyl radicals and Fe^2+^. J. Mater. Chem. 22, 1477–1481 (2012).

[b21] Zhang L. P. *et al.* Fenton reaction-triggered colorimetric detection of phenols in water samples using unmodified gold nanoparticles. Sens. Actuat. B 225, 593–599 (2016).

[b22] Hu R. *et al.* An efficient fluorescent sensing platform for biomolecules based on fenton reaction triggered molecular beacon cleavage strategy. Biosens. Bioelectron. 41, 442–445 (2013). 23062552 10.1016/j.bios.2012.09.013

[b23] Moye A. L. *et al.* Telomeric G-quadruplexes are a substrate and site of localization for human telomerase. Nat. Commun. 6, 7643, 10.1038 /ncomms8643 (2015). 26158869 10.1038/ncomms8643 PMC4510649

[b24] Aizen R. *et al.* G-Quadruplex-Stimulated optical and electrocatalytic DNA Switches. Small 11, 3654–3658 (2015). 25903041 10.1002/smll.201403794

[b25] Hao Y. L. *et al.* Amplified colorimetric detection of mercuric ions through autonomous assembly of G-quadruplex DNAzyme nanowires. Biosens. Bioelectron. 52, 261–264 (2014). 24060975 10.1016/j.bios.2013.08.034

[b26] Xu L. J. *et al.* Turn-on and label-free fluorescence detection of lead ions based on target-induced G-quadruplex formation. Chem. Commun. 51, 8165–8168 (2015). 10.1039/c5cc01590a 25872736

[b27] Liu M. *et al.* A SERS/fluorescence dual-mode nanosensor based on the human telomeric G-quadruplex DNA: Application to mercury(II) detection. Biosens. Bioelectron. 69, 142–147 (2015). 25725462 10.1016/j.bios.2015.02.009

[b28] Li Y. *et al.* A novel electrochemical DNA biosensor based on HRP-mimicking hemin/G-quadruplex wrapped GOx nanocomposites as tag for detection of *Escherichia coli* O157:H7. Biosens. Bioelectron. 63, 1–6 (2015). 25048446 10.1016/j.bios.2014.07.012

[b29] Shi L. J. *et al.* A label-free hemin/G-quadruplex DNAzyme biosensor developed on electrochemically modified electrodes for detection of a HBV DNA segment. RSC Adv. 5, 11541–11548 (2015).

[b30] Chen Q. G. *et al.* An enzyme-free and label-free fluorescent biosensor for small molecules by G-quadruplex based hybridization chain reaction. Talanta 138, 15–19 (2015). 25863365 10.1016/j.talanta.2015.02.002

[b31] Xue Q. W., Wang L. & Jiang W. A novel label-free cascade amplification strategy based on dumbbell probe-mediated rolling circle amplification-responsive G-quadruplex formation for highly sensitive and selective detection of NAD^+^ or ATP. Chem. Commun. 49, 2640–2642 (2013). 10.1039/c3cc39064k 23431564

[b32] Lin Y. P. *et al.* Non-enzymatic sensing of hydrogen peroxide using a glassy carbon electrode modified with a nanocomposite made from carbon nanotubes and molybdenum disulphide. Microchim. Acta 182, 1803–1809 (2015).

[b33] He H. Z. *et al.* G-quadruplexes for luminescent sensing and logic gates. Nucleic Acids Res. 41, 4345–4359 (2013). 23435319 10.1093/nar/gkt108 PMC3632106

[b34] Ma D. *et al.* Simple DNA-based logic gates responding to biomolecules and metal ions. Chem. Sci. 4, 3366–3380 (2013).

[b35] Leung C. *et al.* Luminescent detection of DNA-binding proteins. Nucleic Acids Res. 40, doi:10.1093 (2012). 10.1093/nar/gkr763 PMC3273792 21967849

[b36] Wang M. *et al.* Conjugating a groove-binding motif to Ir(III) complex for the enhancement of G-quadruplex probe behavior. Chem. Sci. 7, 2516–2523 (2016). 10.1039/c6sc00001k PMC5477052 28660021

[b37] Ma D. *et al.* Group 9 organometallic compounds for therapeutic and bioanalytical applications. Acc. Chem. Res. 47, 3614–3631 (2014). 25369127 10.1021/ar500310z

[b38] Leung K. H. *et al.* Label-free luminescence switch-on detection of hepatitis C virus NS3 helicase activity using a G-quadruplex-selective probe. Chem. Sci. 6, 2166–2171 (2015). 10.1039/c4sc03319a PMC5539802 28808523

[b39] Lu L. H. *et al.* Detection of nicking endonuclease activity using a G-quadruplex-selective luminescent switch-on probe. Chem. Sci. 5, 4561–4568 (2014).

[b40] Liu J. *et al.* Iridium(III) complex-coated nanosystem for ratiometric upconversion luminescence bioimaging of cyanide anions. J. Am. Chem. Soc. 133, 15276–15279 (2011). 21892822 10.1021/ja205907y

[b41] Yu M. *et al.* Cationic iridium(III) complexes for phosphorescence staining in the cytoplasm of living cells. Chem. Commun. 2115–2117 (2008). 10.1039/b800939b 18438486

[b42] Ma D. L. *et al.* A luminescent cocaine detection platform using a split G-Quadruplex-selective iridium(III) complex and a three-Way DNA junction architecture. ACS Appl. Mater. Interfaces 7, 19060–19067 (2015). 26284502 10.1021/acsami.5b05861

[b43] Xiong C. *et al.* Sensitive detection of H_2_O_2_ and H_2_O_2_-related reactant with Ru(bipy)_2_(7,8-dimethyl-dipyridophenazine)^2+^ and oligodeoxyribonucleotide. Analyst 137, 4428–4434 (2012). 22893889 10.1039/c2an35519a

[b44] Rai P., Wemmer D. E. & Linn S. Preferential binding and structural distortion by Fe^2+^ at RGGG-containing DNA sequences correlates with enhanced oxidative cleavage at such sequences. Nucleic Acids Res. 33, 497–510 (2005). 15659581 10.1093/nar/gki192 PMC548341

[b45] Tuukkanen S. *et al.* Dielectrophoresis of nanoscale double-stranded DNA and humidity effects on its electrical conductivity. Appl. Phys. Lett. 87, 183102, 10.1063/1.2117626 (2005).

[b46] Cai L. T., Tabata H. & Kawai T. Self-assembled DNA networks and their electrical conductivity. Appl. Phys. Lett. 77, 3105–3106 (2000).

[b47] Wang M. *et al.* Label-free luminescent detection of LMP1 gene deletion using an intermolecular G-quadruplex-based switch-on probe. Biosens. Bioelectron. 70, 338–344 (2015). 25840020 10.1016/j.bios.2015.03.047

[b48] Lu L. H. *et al.* Label-Free luminescent switch-on probe for ochratoxin a detection using a G-quadruplex-selective iridium(III) complex. ACS Appl. Mater. Interfaces 7, 8313–8318 (2015). 25836665 10.1021/acsami.5b01702

[b49] Zhu J., Zhang L. & Wang E. Measurement of the base number of DNA using a special calliper made of a split G-quadruplex. Chem. Commun. 48, 11990–11992 (2012). 10.1039/c2cc36693b 23133833

[b50] Guo S., Xu L. & Xu B. A ternary nanocomposite electrode of polyoxometalate/carbon nanotubes/gold nanoparticles for electrochemical detection of hydrogen peroxide. Analyst 140, 820–826 (2015). 25431885 10.1039/c4an01734j

[b51] Zhang Y., Liu Y. & He J. Electrochemical behavior of graphene/Nafion/Azure I/Au nanoparticles composites modified glass carbon electrode and its application as nonenzymatic hydrogen peroxide sensor. Electrochim. Acta 90, 550–555 (2013).

[b52] Hong J., Yang W. & Zhao Y. Catalase immobilized on a functionalized multi-walled carbon nanotubes–gold nanocomposite as a highly sensitive bio-sensing system for detection of hydrogen peroxide. Electrochim. Acta 89, 317–325 (2013).

[b53] Huang K., Niu D. & Liu X. Direct electrochemistry of catalase at amine-functionalized graphene/gold nanoparticles composite film for hydrogen peroxide sensor. Electrochim. Acta 56, 2947–2953 (2011).

[b54] Roushani M., Bakyas K. & Dizajdizi B. Development of sensitive amperometric hydrogen peroxide sensor using a CuNPs/MB/MWCNT-C_60_-Cs-IL nanocomposite modified glassy carbon electrode. Mat. Sci. Eng. 64, 54–60 (2016). 10.1016/j.msec.2016.03.078 27127028

[b55] Gu T. & Hasebe Y. Peroxidase and methylene blue-incorporated double stranded DNA–polyamine complex membrane for electrochemical sensing of hydrogen peroxide. Anal. Chim. Acta 525, 191–198 (2004).

[b56] Lin X., Chen J. & Chen Z. Amperometric biosensor for hydrogen peroxide based on immobilization of horseradish peroxidase on methylene blue modified graphite electrode. Electroanal. 12, 306–310 (2000).

[b57] Lin K., Yin C. & Chen S. An electrochemical biosensor for determination of hydrogen peroxide using nanocomposite of poly(methylene blue) and FAD hybrid film. Sens. Actuat. B 157, 202–210 (2011).

[b58] Ma D. L. *et al.* A luminescent cocaine detection platform using a split G-Quadruplex-selective iridium(III) complex and a three-way DNA junction architecture. ACS Appl. Mater. Interfaces 7, 19060–19067 (2015). 26284502 10.1021/acsami.5b05861

[b59] Li Z. B. *et al.* Enhanced electrochemical recognition of double-stranded DNA by using hybridization chain reaction and positively charged gold nanoparticles. Biosens. Bioelectron. 74, 687–690 (2015). 26208173 10.1016/j.bios.2015.06.070

